# Patient-reported outcome measures in pediatric asthma care: using theoretical domains framework to explore healthcare providers’ perceptions

**DOI:** 10.1186/s41687-022-00494-3

**Published:** 2022-08-19

**Authors:** Sumedh Bele, Sarah Rabi, Muning Zhang, Elizabeth Oddone Paolucci, David W. Johnson, Hude Quan, Maria J. Santana

**Affiliations:** 1 Department of Pediatrics, Cumming School of Medicine, University of Calgary, Calgary, Canada; 2 Department of Community Health Sciences, Cumming School of Medicine, University of Calgary, Calgary, Canada; 3 Patient Engagement Team, Alberta SPOR Support Unit, Alberta, Canada; 4 Bachelor of Sciences Program, Queen’s University, Kingston, Canada; 5 Bachelor of Health Sciences Program, Cumming School of Medicine, University of Calgary, Calgary, Canada; 6 Department of Surgery, Cumming School of Medicine, University of Calgary, Calgary, Canada; 7 Alberta Health Services Maternal, Newborn, Child and Youth Strategic Clinical Network, Calgary, Canada; 8 Department of Physiology and Pharmacology, Cumming School of Medicine, University of Calgary, Calgary, Canada; 9 Knowledge Translation Team, Alberta SPOR Support Unit, Alberta, Canada; 10 Data and Services Team, Alberta SPOR Support Unit, Alberta, Canada

**Keywords:** Patient-reported outcome measures (PROMs), Asthma, Pediatrics, Theoretical domains framework, Qualitative study

## Abstract

**Background:**

Patient-reported outcome measures (PROMs) play an important role in promoting and supporting patient and family-centered care. Implementing interventions like PROMs in routine clinical care require key stakeholders to change their behavior. The aim of this study was to utilize the Theoretical Domains Framework (TDF) to identify barriers and enablers to the implementation of PROMs in pediatric outpatient asthma clinics from healthcare providers’ perspective.

**Methods:**

This TDF-guided qualitative descriptive study is part of a larger multi-phase project to develop the KidsPRO program, an electronic platform to administer, collect, and use PROMs in pediatrics. Semi-structured qualitative interviews were conducted with 17 participants, which included pediatricians, nurses, allied health professionals and administrative staff from outpatient asthma clinics. All the interviews were transcribed, deductively coded, inductively grouped in themes, and categorized into barriers and enablers.

**Results:**

We identified 33 themes within 14 TDF domains, which were further categorized and tabulated into 16 barriers and 17 enablers to implementing PROMs in asthma clinics. Barriers to behavioral change were attributed to personal, clinical, non-clinical, and other system-level factors; they ranged from limited awareness of PROMs to language barriers and patient’s complex family background. Enablers ranged from a personal commitment to providing patient and family-centered care to administering PROMs electronically.

**Conclusion:**

This implementation of science-based systematic inquiry captured the complexity of PROMs implementation in pediatric outpatient clinical care for asthma. Considering the consistency in barriers and enablers to implementing PROMs across patient populations and care settings, many findings of this study will be directly applicable to other pediatric healthcare settings.

**Supplementary Information:**

The online version contains supplementary material available at 10.1186/s41687-022-00494-3.

## Introduction

The Patient- and family-centered care (PFCC) model is an emerging approach to planning, delivering, and evaluating healthcare, grounded in mutually beneficial partnerships among health care providers, patients, and families [1]. PFCC aims to incorporate and improve all child health and well-being dimensions by engaging individual patients and their family caregivers in co-designing care [1, 2]. To deliver, improve and sustain PFCC, it is crucial to empower children, families, and communities to identify their self-reported outcomes and experiences with the care received [3]. Patient-reported outcome measures (PROMs) play an important role in promoting and supporting PFCC [4, 5].

PROMs are standardized validated questionnaires that capture important aspects of patients’ symptoms, treatment effects, psychological and social impacts, and overall health-related quality of life (HRQOL) [6, 7]. Using PROMs in routine pediatric clinical care: (i) promotes communication between patients, families, and healthcare providers; (ii) improves PFCC outcomes while maintaining low health services utilization; (iii) advances the overall health of the patient; and (iv) enriches healthcare quality [5, 8]. However, within pediatric settings, the use of PROMs still lags behind.

Asthma, which is characterized by chronic airway inflammation, is the most common chronic condition in paediatrics [9]. Clinical care for asthma includes routine outpatient clinical appointments to ensure trigger avoidance, education, regular follow-up, and an action plan that relies on symptom management [10]. Also, asthma impacts quality of life, making it the leading cause of school absenteeism among children [11]. Asthma often requires complex care plans and is a leading cause of hospitalization among the pediatric population [10, 11]. A recent systematic review revealed that using PROMs in routine clinical pediatric care promotes communication between patients, families, and clinicians, improves person-centered outcomes while maintaining low health services utilization, and enhances the patient experience [12, 13]. Thus, implementing PROMs in routine asthma care can facilitate the clinical management of complex chronic clinical care and improve patient quality care outcomes.

As with any intervention, the implementation of PROMs in routine clinical care requires key stakeholders to change their behavior. Therefore, it is important to understand barriers and enablers to changing those behaviours. Theoretically directed research is essential to systematically understand how, why, and under what conditions implementation science techniques facilitate successful implementation of PROMs [14]. However, the use of a robust systematic implementation science-based approach to understanding barriers and enablers in implementing PROMs in pediatric clinical asthma care is scarce. Theoretical Domains Framework (TDF) is one of the frameworks developed for implementation research to identify influences on health professional behavior related to executing evidence-based recommendations such as PROMs [15, 16]. Thus, our study aims to address this research gap by using TDF to understand barriers and enablers to the implementation of PROMs in pediatrics from a healthcare providers’ perspective.

## Methods

This qualitative study is part of a larger multi-phase project, the KidsPRO program, which is an electronic platform to administer, collect, and graphically represent PROMs data to be shared with patients, their family caregivers, and clinicians supporting the implementation of PROMs in pediatric clinical care [17]. The Conjoint Health Research Ethics Board at the University of Calgary approved this study (REB18-0564). Additionally, administrative approval for this project was obtained from Alberta Health Services (AHS).

### Study design

TDF guided this qualitative descriptive study design. The progression from theory-based investigation to intervention design provides a theoretical basis to understanding potential barriers for the slow uptake of evidence into practice, and the enablers that may influence the phenomenon [18]. TDF consists of 84 component constructs refined into 14 theoretical domains (Table 3) [15, 16]. It helps to describe the empirical phenomenon (implementation of PROMs) by fitting them into a set of categories.

### Study site and recruitment

The study sites included outpatient asthma clinics at the Alberta Children’s Hospital (ACH) and Calgary's urban asthma community clinics. ACH is a teaching hospital affiliated with the University of Calgary and is one of the largest tertiary level pediatric hospitals in Canada. The ACH outpatient clinic provides care for approximately 30 patients per week and similar average numbers at the community clinics. The clinician team comprises of eight respirologists, 11 pediatricians, 16 nurses and educators, and 12 allied health professionals (two social workers, one clinical psychologist, and nine technicians from the pulmonary function test laboratory).

We used a stratified purposive sampling strategy to recruit a diverse sample of clinicians from the ACH outpatient asthma and community clinics. Our sample included general pediatricians, pediatric respirologists, nurses, allied health providers, and clinic staff. The diversity among participants helped us understand perceived barriers and enablers to implementing PROMs in pediatric asthma clinical care. Potential participants were identified by leaders of the ACH asthma clinic, and subsequently directly approached via email by the research team.

### Materials and data collection

The interview guide was developed based on TDF and included between two and four questions for each of the 14 domains of the TDF (see Additional file 1: Appendix I). Before each interview, the interviewer provided more information about the PROMs by sharing the Pediatric Quality of Life Inventory™ (PedsQL™) [19] Version 4.0 Generic Core Scales and PedsQL™ Asthma Specific Module, and explained details of the KidsPRO program [17]. PedsQL™ requires about 5 min to complete and similar time is required to complete its asthma module. More information about PedsQL is provided in Table 1.KidsPRO is an ehealth solution that supports and facilitates integration of PROMs into routine pediatric clinical care. KidsPRO was developed as a standalone program with abilities to be incorporated into the EMR system. However, at the time of this study KidsPRO was not integrated within the local EMR system. The KidsPRO application will be available to patients and family caregivers on mobile devices, tablets, and desktop computers, which enables patients and families to complete self-reported measures from home prior to visiting the clinics. KidsPRO generates results that are graphically presented and available to patients, family caregivers and their providers at the time of their clinic consultation.

**Table 1:** Description of PedsQL™ questionnaires

Measure	Description
Pediatric Quality of Life Inventory™ (PedsQL™) 4.0 Generic Measure	A 23-item generic score scale to measure HRQOL in healthy children and adolescents and those with acute and chronic health conditions
	Consists of four domains:
	1. Physical functioning
	2. Emotional functioning
	3. Social functioning
	4. School functioning
Pediatric Quality of Life Inventory™ (PedsQL™) 3.0 Asthma Module	An asthma specific 28 items score scale to complement Generic Core scale
	Consists of four domains:
	1. Asthma
	2. Treatment
	3. Worry
	4. Communication

As our data collection efforts were impacted by the Covid-19 pandemic, a mix of in-person and virtual interview meetings were conducted by a single interviewer (SB). Interviews ranged between 26 and 55 min in length. All the interviews were audio-recorded and transcribed verbatim.

### Data analysis

All the transcribed transcripts were imported into NVivo 12 [20] to code, organize, and manage the data. Before analyzing all the data, two randomly selected transcripts were coded independently by three reviewers of the research team (SB, SR, MZ) to develop a codebook.Consensus on the codebook was reached through discussion. Later, a single reviewer (SB) coded all the remaining transcripts using this codebook.

Data were analyzed in three consecutive steps: (i) a directed content analysis approach [21] was used to systematically code and categorize similar statements in each of the 14 domains. If any statements were relevant to more than one domain, then they were cross-indexed to multiple domains; (ii) an inductive approach was applied to combine similar statements into themes within the 14 TDF domains; and (iii) themes were further categorized and tabulated into barriers and enablers. Quotations illustrating core statements were used to support barriers, enablers, or major theme(s) in each domain.

## Results

We interviewed 17 clinicians and administrators, including four working at the community outpatient clinics and 13 working at the ACH outpatient asthma clinics, which comprised half of the full-time staff at the asthma clinics (Table 2). After 15 interviews, we had already reached thematic saturation, however, we still interviewed two more clinicians who agreed to participate.

**Table 2:** Characteristics of study participants (n = 17)

Characteristics	Category	Participants (n = 17)
Gender	Male	4 (24%)
	Female	13 (76%)
Site	Alberta Children’s Hospital Clinics	13 (76%)
	Community Clinics	4 (24%)
Position	Administrator	3 (18%)
	Hospital pediatrician	4 (24%)
	Pediatric respirologist	3 (18%)
	Certified Respiratory Educator	3 (18%)
	Other allied health professionals (Psychologist, social worker, and Nurse)	4 (24%)

All the interview transcripts were deductively coded, inductively grouped into themes, and categorized into barriers and enablers (Table 3). Table 4 shows the categorization of perceived barriers and enablers.

**Table 3:** Sub-themes identified in all 14 domains of Theoretical Domains Framework

Domain (Definition)	Sub-themes	Quotes
**1. Knowledge** (An awareness of the existence of something)	Limited awareness	*“No, never heard of it.”* (P03, Certified Respiratory Educator) *“I've attended a few presentations that [PI] made and you've made that you’ve come and presented in our Education Day.”* (P01, Administrator)
**2. Skills** (An ability or proficiency acquired through practice)	Communication skills	*I feel like all health care providers have general communication skills that would allow them to ask these questions.”* (P15, Allied health professional) *“I think that there should be a skill, in terms of teaching the families how to fill this out.……. just a basic training in terms of how to present it to the families, how to frame it.”* (P09, Pediatrician)
	Data interpretation skills	*“in terms of the data interpretation, or how you use that in your clinical practice.”* (P09, Pediatrician) *“I think that level of understanding of “what are the questions?” “what are the variability between different participants answers?” and therefore “what would que you to potentially pursue a particular area?” I think would be helpful.”* (P04, Pediatrician)
	Other skills	*“I would need like, technology skills to be able to view the results in an efficient manner prior to the clinic so nothing more clinically but to be understanding on how to incorporate into practice and probably some technical skills.”* (P11, Pediatric Respirologist) *“I think they have to be able to incorporate this piece of information into their scheduling for the clinic.”* (P01, Administrator)
**3. Social/professional role and identity** (A coherent set of behaviours and displayed personal qualities of an individual in a social or work setting)	Providing patient and family-centered care	*“it (PROMs) would give a good understanding of how kids are feeling, and coping, how they're doing in all aspects of their lives and their parents perceptions of how they're doing as well. And that can better help the team understand different challenges that might come up with their medical management.”* (P01, Administrator) *“So, this is where PROMs are important in that you have to align your goals with theirs (patients).”* (P13, Pediatric Respirologist)
	Lack of guidelines from professional organizations	*“I would say I don’t- I am not aware of anything formal that is out there.”* (P04, Pediatrician)
**4. Beliefs about capabilities** (Acceptance of the truth, reality, or validity about an ability, talent, or facility that a person can put to constructive use)	Resistance to change culture	*“I think the devil is always in the details and requires culture change, you know, changing processes and then sustaining that process overtime until it becomes part of what we do all the time.”* (P04, Pediatrician) *“Some are going to embrace it; some are going to see the value and others are gonna “there's nothing wrong with the way I'm doing it.”* (P02, Pediatric Respirologist)
	PROMs as standardization tools	*“in asthma you know everybody likes to do things their own way. Really hard with something that is actually helping people standardize what they're doing.”* (P07, Administrator) *“Just because every time a patient comes to the clinic, they may be seeing different physician, so standardized approach is difficult to do for anything that we add in our clinics.”* (P11, Pediatric Respirologist)
	Confidence in self-ability	*“I think it would be it would be easy if the information is provided beforehand or at the time of the arrive at the clinical appointment. In an easy way for the healthcare provider to view the result.”* (P10, Allied health professional) *“I think, pretty confident. Yeah, I think we do it all the time in an informal way, but I think it's actually, it's probably going to be a nice add on to our history taking anyway, because we probably should be.”* (P09, Pediatrician)
**5. Optimism** (The confidence that things will happen for the best or that desired goals will be attained)	Optimism about positive impact of PROMs	*“in general, I would feel confident that it could improve quality of life for families.”* (P16, Allied health professional) *“they (patients and families) think that its an ongoing care even if they are at home, they can answer the questionnaire and really think about it, come to the clinic with, with all the information that we need. So, in that way improve patient care.”* (P11, Pediatric Respirologist)
**6. Beliefs about consequences** (Acceptance of the truth, reality, or validity about outcomes of a behaviour in a given situation)	PROMs for comprehensive health	*“I think the biggest benefit is improving the comprehensive health of the patient. Moving beyond just dealing with the chief complaint.”* (P07, Administrator) *“those psychosocial things that we don't normally investigate but are very important things that may have links with other places…… I mean I think big picture using a tool like that (PROM) really does add to the Comprehensive Assessment of a patient.”* (P04, Pediatrician)
	Optimizing healthcare delivery	*“I think it really helps people think about their symptoms and their expectations prior to when they come for appointment.”* (P03, Certified Respiratory Educator) *“Results are given prior to seeing the patient, and they give you an idea of what things around and they have kind of didn't we, we are you going to address things.”* (P12, Pediatrician)
	Benefits of using PROMs outweighs harms	*“I think that as long as the “how” is done well, then the benefits should definitely well outweigh the harms.”* (P17, Allied health professional) *“I think the benefits definitely outweigh the risks for our patients, knowing a bit more about them ahead of time I think is a huge benefit.”* (P08, Certified Respiratory Educator)
	Lack of clear processes and strategies	*“because when someone flags, you know, their top three concerns, I think it would be- it's not professional for us not to deal with them. And so, we better have some strategies or some plans in place for what do we do when families say this?”* (P02, Pediatric Respirologist)
	Potential negative consequences	*“we obviously we don’t want to overburden families, especially those with a child with chronic illness.”* (P16, Allied health professional) *“you have a scoring system like this, sometimes you don't want to fail so people will give you the answer that you- that they think you want, and it may not be the truthful answer.”* (P03, Certified Respiratory Educator) *“I think that we might end up identifying things that are less directly associated with that, with their medical condition potentially. And while that might be a very good thing, because it might influence their care in that area, (but) it could also divert time and energy into somethings that maybe aren’t directly as related, let’s say in this case to their asthma.”* (P04, Pediatrician)
**7. Reinforcement** (Increasing the probability of a response by arranging a dependent relationship, or contingency, between the response and a given stimulus)	Incentives	*“I think it might make our job a wee bit easier.”* (P05, Certified Respiratory Educator) *“our main incentive is to always try to better understand how we can meet a family’s needs while trying to improve their child’s trajectory of their chronic illness, asthma in this case.”* (P06, Administrator)
	Mixed perceptions with time	*“barrier. I think that's the biggest thing is, how much extra time would it take for you to go through it. Before the clinic or during the clinic.”* (P11, Pediatric Respirologist) *“I know people always say “oh, it's a barrier of time,” well really, it's no barrier of time at all, you're doing the work anyway. In fact, this may actually improve your time because some- some of these- this is all answered for you, so it would- it will be really interesting.”* (P03, Certified Respiratory Educator) *“It (PROMs) may keep it (time) neutral; it may raise some areas that take more time to address it. But I think they do more comprehensive job on providing care so maybe depends on how you look at it.”* (P12, Pediatrician)
	Motivation for clinicians	*“(it depends on) how easy you're going to make it for my team members when they come into a patient appointment to be able to see those results so that they can frame their conversation.”* (P02, Pediatric Respirologist) *“I think it'd be pretty easy to use. I think uptake would be dependent on how much the patients buy into it.”* (P14, Pediatrician)
	Motivation for patients and families	*“what the parents and families and kids feel they're going to get out of it is also another concern, because if they don't feel it's relevant or important, then they're not going to do it.”* (P01, Administrator)
**8. Intentions** (A conscious decision to perform a behaviour or a resolve to act in a certain way)	High importance	*“I think we should give it a 10 (out of 10).”* (P03, Certified Respiratory Educator) *“I would say the PROMs are at the 8 to 10 scale.”* (P01, Administrator)
**9. Goals** (Mental representations of outcomes or end states that an individual wants to achieve)	Compatibility	*“I think I would still continue doing what I was doing, but I think it would help to inform maybe where to focus on.”* (P15, Allied health professional) *“It's definitely compatible our current practice because we're very electronic based on the fact thar we have a new electronic system that's gonna be implemented in the next year.”* (P01, Administrator)
**10. Memory, attention and decision processes** (The ability to retain information, focus selectively on aspects of the environment and choose between two or more alternatives)	Language barriers	*“Probably just the language barriers. You know we create so many of our documents so many of our questionnaires are for the English-speaking people at a certain grade level. But not everybody is English speaking or at that grade level.”* (P14, Pediatrician) *“And things like language barrier and things like that we have some strategies for using interpreters and that kind of thing, which mitigate things to some degree.”* (P10, Allied health professional)
	Technological barriers	*“I think it may be difficult if the patient doesn't have electronic access or doesn't have a cell phone or an iPad or we don't have iPads available, that's going to be another impediment.”* (P01, Administrator) *“I think having access to maybe the technology. You know, if they go online and their computer is not up to date, you can’t open it, you can't get the password, like I'm not going to spend five minutes trying to do that as a patient.”* (P13, Pediatric Respirologist)
	Complex family background	*‘We have lots of kids that live in multiple households -mom, dad, grandparent, aunt- just like [name] was saying, so it gets complicated. Where- who's giving you the insight, right?”* (P03, Certified Respiratory Educator) *“I think kind of high conflict family, where there's parental conflict, or challenges between parents, I think certainly families that are maybe, generally, quote unquote non compliant with medical care.”* (P15, Allied health professional)
**11. Environmental context and resources** (Any circumstance of a person’s situation or environment that discourages or encourages the development of skills and abilities, independence, social competence, and adaptive behaviour)	Challenges of working within larger system	*“Challenge is more working…. working within the system, like AHS (Alberta Health System) that can make it more challenging for you with an existing system.”* (P12, Pediatrician) *“I think, it would be pretty easy if we had the frontline nurse get them set up with that, ahead of time, even have iPads (at the clinics) and results could just be automatically uploaded to EMR. I think that would be pretty slick.”* (P09, Pediatrician)
	Acuity of the patient	*“Obviously, if a child is acutely unwell at the time, which does sometimes happen, it might be inappropriate potentially to veer away from trying to acutely address the problem at hand, where the child might have a deteriorating condition or might need to have acute management and really that has to be the focus of the time that we spend that time on, so I could see that playing a role. That would probably be the main situation where it would be not possible to kinda go there (PROMs).”* (P04, Pediatrician) *“acuity, acute status of patients would be one of the clinical factors associated with clinical environment (impacting use of PROMs).”* (P13, Pediatric Respirologist)
	Disruption to clinical workflow	*“if we were already running way behind and had particular time constraints for patients in clinic it might be a choice that we have to decide what we’re going to address that day.”* (P07, Administrator) *“I'm doing now is using our pulmonary function testing results and discussion to family and physical examination I think that's our core and key aspect and if it's starting to, you know, interfere with that piece I may not use it on a regular basis.”* (P13, Pediatric Respirologist)
**12. Social influences** (Those interpersonal processes that can cause individuals to change their thoughts, feelings, or behaviours)	Influence of leaders and team members	*“It kind be of very high level; I think behind being positive idea. "yes this makes sense" there's been a very high level.”* (P12, Pediatrician) *“Multiple people, like clinic lead, Alberta Health Services supporting it, buy-in from all the support staff. Nursing—that’s a big one.”* (P09, Pediatrician)
	Patients’ and families’ emotions	*“Because parents, that’s who influenced us, that’s how we got where we were, our parents influenced us, parents influence children.”* (P03, Certified Respiratory Educator) *“If the patient and family are not routinely doing them, then it’s less likely that we’ll be successful in integrating them because it will be more kind of one-off instead of the usual, so that’s really important.”* (P04, Pediatrician)
**13. Emotion** (A complex reaction pattern, involving experiential, behavioural, and physiological elements, by which the individual attempts to deal with a personally significant matter or event)	Excitement	*“I think it’s always kind of exciting to see us move into a more patient and family focused direction.”* (P14, Pediatrician) *“excited sounds kind of, like, sort of sounds like a personal thing rather than a professional thing but I think our group is excited about doing new things that are proven to be better and challenging our skills.”* (P05, Certified Respiratory Educator)
**14. Behavioural regulation** (Anything aimed at managing or changing objectively observed or measured actions)	Electronic PROMs	*“And I think our population and our parents are keen to use electronic measures as opposed to paper questionnaires.”* (P06, Administrator) *“think having it online is helpful, especially for teens, they’re more likely to do things online, so then they don't have to worry about bringing the paper with them, so you have tried to take away that barrier.”* (P10, Allied health professional)
	Engagement with stakeholders at the asthma clinic	*“So having communication with all the stakeholders from administration to pediatric division, to respiratory division, to allied health division, to the family division, to patient division, we have to make sure we have all the right players involved in decision making all the way along that we communicate effectively.”* (P01, Administrator) *“I think if you can bring them a compelling story that resonates to them about how this can make a difference, if they can see it in action and if they can hear some feedback from a family that says this made all the difference in the world.”* (P02, Pediatric Respirologist)

**Table 4:** Barriers and enablers to implementation of PROMs in pediatric outpatient asthma clinics

Domain	Barrier	Enabler
Knowledge	Limited awareness	
Skills	Data interpretation skills	Communication skills
Social/professional role and identity	Lack of guidelines from professional organizations	Willingness to Provide patient and family-centered care
Beliefs about capabilities	Resistance to change culture	As a standardization tool Ease of integrating PROMs Confidence in self-ability
Optimism		Optimism about positive impact of PROMs
Beliefs about consequences	Lack of clear processes and strategies Potential negative consequences	To deliver comprehensive healthcare To optimize healthcare delivery Benefits of using PROMs outweighs harms
Reinforcement	Perceptions with time (increases appointment time) Specific motivations for clinicians Specific motivations for patients and families	Perceptions with time (decreases appointment time) Incentives
Intentions		High importance
Goals		Compatibility
Memory, attention and decision processes	Language barriers Technological barriers Complex family background	
Environmental context and resources	Challenges of working within larger system Acuity of the patient Disruption to clinical workflow	
Social influences	Patient and families’ emotions	Influence of leaders and team members
Emotion		Excitement among healthcare providers
Behavioural regulation		Electronic PROMs Engagement with stakeholders at the asthma clinic

## Domain 1: knowledge

### Theme: limited awareness

Most participants had not heard of the term “Patient-reported Outcome Measures”, but they were aware of surveys, either created by their own teams or administered by their health system. Some of the participants had heard of PROMs at academic conferences or through scientific literature and presentations made by our research team as part of stakeholder engagement activities.

## Domain 2: skills

### Theme 1: communication skills

Eleven respondents acknowledged that they already had communication skills needed to discuss concerns raised by PROMs. However, participants suggested the need for additional skills to teach families the purpose of collecting information and understanding the PROMs results.

### Theme 2: data interpretation skills

Clinicians felt that they would need some training in interpreting PROM results, so as to use them accurately and to assist them in making the most appropriate clinical decisions for their patients.

### Theme 3: other skills

A few respondents also identified the need to receive skills in technology, especially around administering PROMs and accessing the results. Moreover, scheduling and time management skills to incorporate PROM information within routine clinical workflow were listed as possible areas for respondents to receive training.

## Domain 3: social/professional role and identity

### Theme 1: providing patient and family-centered care

Participants felt that PROMs would help them with a holistic understanding of patients’ and families’ needs, including psychosocial aspects impacting their health status, which is vital for the comprehensive assessment of their patients. Participants also highlighted PROMs’ role in patient empowerment, which involves capturing patients’ and their family member’s perspectives in a standardized manner.

### Theme 2: lack of guidelines from professional organizations

All participants were asked if they were aware of any guidelines or had received training on using PROMs from their professional organizations. Although clinicians underscored that providing patient and family-centered care is encouraged by professional organizations, they had not received any formal training and were unaware of any practice guidelines on this topic.

## Domain 4: beliefs about capabilities

### Theme 1: resistance to change the culture

According to participants, successful implementation of PROMs in their clinics would also depend on culture change at the clinics. However, many participants cautioned about the potential resistance in changing the current work cultures and processes.

### Theme 2: PROMs as a standardized tool

Participants considered PROMs to be a valuable tool in standardizing the care provided by different healthcare providers at the asthma clinics. Yet, it was noted that since each healthcare provider has their own way of providing care and patients typically see different healthcare providers at every visit, it would be challenging for everyone to use PROMs in the same way.

### Theme 3: ease of integrating PROMs

When answering the question regarding ease of integrating PROMs, participants believed that it would be easier if the PROMs were administered before patient appointments, as this would offer them timely and straightforward access to the PROM results.

### Theme 4: confidence in self-ability

All the interviewed frontline healthcare providers exhibited confidence in using PROMs as part of their clinical care.

## Domain 5: optimism

### Theme 1: optimism about the positive impact of PROMs

Participants largely believed that implementing PROMs in clinical care would improve patient care. Many participants also affirmed that PROMs would not drastically change the current practice of providing care, but rather, would enhance it. According to one participant, PROMs would only add value if an appropriate PROM were used; otherwise, it would just be “extra work”.

## Domain 6: beliefs about consequences

### Theme 1: PROMs for delivering comprehensive healthcare

When asked about the benefits of using PROMs in clinical care, participants suggested that using PROMs would help them understand the overall impact of the clinical condition on the patient and provide comprehensive care aligned with patients’ goals.

### Theme 2: optimizing healthcare delivery

Clinicians felt that using PROMs would optimize healthcare delivery by helping patients and families pre-think their expectations for their appointments, as well as aid clinicians in better planning appointments based on the issues raised through PROMs. Additionally, PROMs were considered a useful tool in collecting standardized information from patients and families to ensure that clinicians could compare the aggregated results between the clinics and improve care delivery, especially for those with higher identified needs.

### Theme 3: benefits of using PROMs outweighs the harms

When asked about whether the benefits of implementing PROMs outweigh the harms or vice versa, all the participants unanimously agreed that the benefits would outweigh the harms.

### Theme 4: lack of clear processes and strategies

Participants pointed out that PROMs would help them recognize their patients’ psychosocial concerns, but they might feel helpless without clear strategies to deal with those concerns. Therefore, having clear guidelines and standard processes was considered necessary.

### Theme 5: potential negative consequences

Participants pointed out several potential negative consequences of using PROMs, which included spending more time and energy on issues not directly related to their asthma, disrupting clinics’ workflow, burdening families having children with chronic conditions, teens providing false information if their parents could access their psychosocial domain PROM scores, and patients and families inflating PROM scores if they were perceived as exam scores.

## Domain 7: reinforcement

### Theme 1: incentives

The personal incentive to implement PROMs in asthma clinics listed by participants included a better understanding of their patients’ and families’ needs, providing the best possible care for their patients, increasing professional satisfaction, making their jobs easier by activating patients, and increasing their efficiency.

### Theme 2: mixed perceptions with time

Participants expressed different opinions about the impact of using PROMs on the total duration of the appointment. Some participants believed that using PROMs would unearth more psychosocial concerns, which might require additional time to address those concerns, increasing appointment times. Other participants believed that PROMs would help them pre-ask some of the questions before the appointment, so that communication could then directly focus on the major issues raised by patients through PROMs. Lastly, some participants felt that appointment time would be unchanged because the time required to address additional concerns would be balanced by eliminating some generic questions usually asked during the appointment.

### Theme 3: motivation for clinicians

Non-physician participants emphasized the importance of buy-in from physicians as one of the most critical factors in the successful implementation of PROMs in asthma clinics. When asked about ways to increase buy-in from physicians, participants emphasized the importance of demonstrating the impact and efficiency of PROMs implementation on various outcomes.

### Theme 4: motivation for patients and families

Participants advised that the questionnaires should be short and not create an additional burden on patients and their families. In addition to clinicians, patient and their families should also be motivated to complete PROMs; without their buy-in, it would not be possible to implement PROMs in asthma clinics.

## Domain 8: intentions

### Theme 1: high importance

Participants were asked to rate the importance of implementing PROMs in routine asthma clinical care on a scale of one to ten, where one represented ‘least’ and ten represented ‘very important’. Eight was the median score given by participants. Higher scores on this question showed the perceived importance of implementing PROMs in asthma clinics. Reasons for giving higher scores included the importance of PROMs for patients, getting more information about patients, and curiosity to try a new intervention. Lower scores were mainly associated with skepticism due to participants’ lack of experience using PROMs.

## Domain 9: goals

### Theme 1: compatibility

Fourteen participants felt that the implementation of PROMs in asthma clinics would be highly compatible because the questions asked in PROMs would complement their current history. Additionally, the electronic administration of PROMs was seen as compatible with the incoming province-wide implementation of a new electronic medical record (EMR) system. Still, one participant pointed out that its compatibility would rely on ironing out the logistics of administering, collecting, and sharing the results through the EMR. Another participant raised the worry that PROMs might be incompatible with the current clinical workflow since, currently, they barely get through the main complaint in 30 min appointments.

## Domain 10: memory, attention and decision processes

### Theme 1: language barriers

Nine participants mentioned that language barriers would create challenges to incorporating PROMs in clinical care. The reading skills of the non English-speaking population could create a language barrier. Several suggestions to mitigate this situation were offered, such as translating the questionnaire in multiple languages and including the help of language support systems (e.g., interpreters and language line) through AHS.

### Theme 2: technological barriers

The lack of reliable access to technology was considered an important barrier for lower socioeconomic status families. Moreover, digital illiteracy was also considered a concern for patients and their families who may be unable to complete electronically administered PROMs from home or at the clinics prior to their appointments.

### Theme 3: complex family background

Participants also highlighted that patients’ complex family background such as,living in different households, large families with multiple caregivers, or those experiencing parental conflicts would hamper use of PROMs among these patients. Patients coming with complicated family backgrounds may have substantial psychological concerns beyond asthma, making it challenging to use PROMs for such patients.

## Domain 11: environmental context and resources

### Theme 1: challenges of working within the larger system

Participants pointed out that asthma clinics work in a larger provincial healthcare system, so although their clinics might be keen on implementing PROMs in routine clinical care, the lack of other supporting systems, such as integration within the EMR system, would challenge their implementation.

### Theme 1: acuity of the patient

Participants highlighted that if the patient needed acute care, asking them to complete PROMs or even discussing the PROM results would not be possible.

### Theme 2: disruption to clinical workflow

If using PROMs would lead to workflow disruption or compete with clinicians’ ability to use biological or pulmonary test results, then they would not prioritize the use of PROMs.

## Domain 12: social influences

### Theme 1: influence of leaders and team members

Fifteen participants from ACH asthma clinics denied discussing the use of PROMs in clinical care with their colleagues. However, those from community clinics reported having discussed it with their colleagues, and mentioned that their discussion was very positive towards using PROMs. Participants listed many stakeholders who would influence their decision to use PROMs in clinical care, with clinical leads and managers being considered the most influential. Moreover, pediatricians were also listed as influential for other team members.

### Theme 2: patient and families’ emotions

Patients and their family caregivers’ emotions also carried a significant influence on healthcare providers’ decisions to use PROMs in asthma clinics. Patients and their family members need to complete PROMs, so implementation of PROMs was not considered feasible without their engagement.

## Domain 13: emotion

### Theme 1: excitement

Participants exhibited a mix of emotions towards using PROMs as part of their clinical care. While some participants showed excitement mainly because of PROMs’ ability to provide patient and family-centered care, others remained emotionally neutral towards the prospect of using PROMs. Two participants raised some cautions, such as the potential of increasing workload and potentially uncovering more psychosocial determinants of health, for which they might not be prepared to manage.

## Domain 14: behavioural regulation

### Theme 1: electronic PROMs

While answering this final question, several participants reiterated the advantages of electronic PROMs and suggested keeping them online. Participants also suggested that user testing of the electronic platform with patients and families to ensure its acceptability and simplicity in receiving and filling PROMs would be important for patients’ and families.

### Theme 2: engagement with stakeholders at the asthma clinic

Implementation of an intervention like PROMs in clinical care warrants the involvement of many stakeholders across many divisions in the hospital, so it was suggested to engage the right people at the right time and the right place. Sharing scientific literature and anecdotal stories from patients showing the real-world impact of using PROMs on their health would help in increasing buy-in from clinicians.

### Barriers and enablers to implementation of PROMs

Based on the interviews, we identified 33 Themes within 14 TDF domains, as shown in Table 3 with supporting participant quotes. We further categorized and tabulated these themes into 16 barriers and 17 enablers to implementing PROMs in asthma clinics, as listed in Table 4.

## Discussion

Patient-reported Outcome Measures are increasingly being used in pediatric clinical care due to their ability to capture the patient “voice”, empower patients and families, and facilitate delivery of PFCC [8, 22]. However, there are myriad of challenges associated with their implementation in routine clinical care. We utilized TDF to systematically explore barriers and enablers to implementing PROMs in routine pediatric asthma care. TDF was chosen for this study because it provides a robust theoretical and comprehensive lens to view the cognitive, affective, social, and environmental influences on behavior and covers most of the potential reasons for implementation problems [23].

Seventeen barriers to behavioral change identified in our study were attributed to personal, clinical, non-clinical, and other system-level factors. The barriers such as limited awareness of PROMs and the need for PROMs data interpretation skills underline the role of healthcare systems, educational institutions, and professional organizations to create awareness about the use of PROMs and advance the skills required for frontline clinicians to implement PROMs in clinical care. Outside the clinical environment, language and technological barriers, and patient and family issues were associated with economic, social, and cultural aspects. The motivations for using PROMs might differ for clinicians and patients and their families, so non-alignment of their motivations could create barriers to implementing PROMs. Similarly, the emotional state of patients and families could deter them from completing PROMs and act as one of the barriers.

Among the 17 enablers, clinicians’ commitment to providing patient and family-centered care, excitement, high importance, and optimism about using PROMs to provide comprehensive healthcare was identified as a major enabler. Compatibility of using electronic PROMs with current practice, competency in communication around psychosocial questions, confidence in self-abilities, demonstrate feasibility of implementing PROMs in asthma clinics. Moreover, the perception of PROMs as tools to standardize care across asthma clinics and optimize healthcare delivery underlines the additional uses of PROMs in asthma clinics. Lastly, our team’s engagement with the senior leadership and all the staff at the asthma clinics was considered a major enabler.

AHS is currently rolling out a province wide EMR system. Therefore, the findings of this study will facilitate the integration of PROMs within this EMR system or through the KidsPRO program. Although mitigation of barriers related to clinical workflow, organizational culture and would warrant system-level changes, barriers such as the need for skills (data interpretation, etc.) identified by clinicians, would be utilized to develop user guides for planning the use of PROMs through the KidsPRO program [17]. To mitigate technological barriers, the KidsPRO program will have tablets and support mechanisms at the clinics for patients to complete PROMs at the clinics prior to their appointment [17]. Senior leaders and clinical leads will be presented with the findings of this study to develop a pan-hospital implementation and province-wide scale-up of the KidsPRO program.

Previous systematic reviews had found that healthcare organizations needed to invest time and resources in “designing” the context-specific PROM strategy and reported mixed results on the perceived impact of using PROMs on the average duration of an appointment or consultation [12, 24] corroborating with those study findings. Therefore, future studies should objectively measure the impact of implementing PROMs on the time of appointment. The findings of our study, like the need for professional development and training, including patient-family education, align with the findings from a study exploring stakeholder perspective on clinical implementation of PROMs in pediatric solid organ transplantation [25]. Similarly, barriers such as lack of organizational support to incorporating PROMs into existing workflows has been identified in a previous study [26]. On the other hand, similarities exist between enablers from our study and previous studies. For example, compatibility of PROMs implementation with clinicians' values has been identified as a facilitator [27], which this aligns with one of the enablers identified in our study i.e. willingness to provide patient and family-centered care. Some of the barriers and enablers identified in our study might have been healthcare system and local context specific. But according to a recently published study, barriers and enablers to implementing PROMs are remarkably consistent across patient populations and care settings [14]. Therefore, many of the findings from our study apply to other healthcare settings.

The current Covid-19 pandemic has resulted in school closures and social isolations, which have increased psychosocial stress on children and adolescents [28]. Considering the role of PROMs in capturing the psychosocial concerns of patients, health systems around the world should expedite the implementation of PROMs in routine pediatric clinical care.

### Strengths and limitations

One of the strengths of our study is the diversity in our sample, which included frontline clinicians, allied health professionals, and administrators, who provided diverse views of the barriers and enablers in asthma clinics. The systematic and theoretical domains framework-driven approach to identify potential barriers and enablers is another key strength of this study. The findings of this study must be interpreted with caution, keeping some limitations in mind. For instance, our use of PedsQL™ as an example of a typical PROM might have influenced some responses, especially around psychosocial questions. Also, this study was conducted at a single tertiary academic hospital and community clinics run by a single team, so the results might not be completely transferrable to other healthcare settings.

## Conclusion

The implementation of PROMs in pediatrics is lagging compared to adult populations. This study contributes a comprehensive and systematic inquiry of perceived barriers and enablers to the implementation of PROMs in routine clinical care to the growing body of scientific literature on PROMs in pediatrics. Considering the consistency in barriers and enablers to implementing PROMs across patient populations and care settings, the findings of this study can be translated to other pediatric healthcare settings.

## Supplementary Information

**Additional file 1**. **Appendix 1:** Interview Guide.

## Data Availability

The data that support the findings of this study are available from the corresponding author, MS, upon reasonable request.

## References

[1] Patient-and Family-Centered Care (2021) Institute for Patient-and Family-Centered Care. Published 2021. https://www.ipfcc.org/about/pfcc.html

[2] Hsu C, Gray MF, Murray L, et al (2019) Actions and processes that patients, family members, and physicians associate with patient- and family-centered care. BMC Family Pract 20(1). doi:10.1186/S12875-019-0918-7 10.1186/s12875-019-0918-7 PMC6388493 30803446

[3] Eichner JM, Johnson BH, Betts JM (2012). Patient- and family-centered care and the pediatrician’s role. Pediatrics.

[4] Santana MJ, Manalili K, Jolley RJ, Zelinsky S, Quan H, Lu M (2018). How to practice person-centred care: a conceptual framework. Health Expect.

[5] Santana MJ, Feeny D (2013). Framework to assess the effects of using patient-reported outcome measures in chronic care management. Qual Life Res.

[6] Kingsley C, Patel S (2017). Patient-reported outcome measures and patient-reported experience measures. BJA Educ.

[7] Weldring T, Smith SMS (2013). Patient-reported outcomes (PROs) and patient-reported outcome measures (PROMs). Health Services Insights.

[8] Greenhalgh J, Gooding K, Gibbons E, et al. How do patient reported outcome measures (PROMs) support clinician-patient communication and patient care? A realist synthesis. J Patient-Rep Outcomes. 2018;2. doi:10.1186/S41687-018-0061-6 10.1186/s41687-018-0061-6 PMC6153194 30294712

[9] Hoch HE, Houin PR, Stillwell PC (2019). Asthma in children: a brief review for primary care providers. Pediatr Ann.

[10] Sont JK (1999) How do we monitor asthma control? Allergy 54 Suppl 49(49):68–73. doi:10.1111/J.1398-9995.1999.TB04391.X 10.1111/j.1398-9995.1999.tb04391.x 10422751

[11] Hsu J, Qin X, Beavers SF, Mirabelli MC (2016). Asthma-related school absenteeism, morbidity, and modifiable factors. Am J Prev Med.

[12] Bele S, Chugh A, Mohamed B, Teela L, Haverman L, Santana MJ (2020). Patient-reported outcome measures in routine pediatric clinical care: a systematic review. Front Pediatr.

[13] Bele S, Mohamed B, Chugh A, Haverman L, Santana MJ (2019). Impact of using patient-reported outcome measures in routine clinical care of paediatric patients with chronic conditions: a systematic review protocol. BMJ Open.

[14] Stover AM, Haverman L, van Oers HA (2021). Using an implementation science approach to implement and evaluate patient-reported outcome measures (PROM) initiatives in routine care settings. Qual Life Res.

[15] Cane J, O’Connor D, Michie S (2012). Validation of the theoretical domains framework for use in behaviour change and implementation research. Implement Sci.

[16] Michie S, Johnston M, Abraham C (2005). Making psychological theory useful for implementing evidence based practice: a consensus approach. Qual Saf Health Care.

[17] Bele S, Oddone Paolucci E, Johnson D, Santana M (2019) Integration of patient-reported outcome measures in routine pediatric asthma care using KidsPRO program. In: Quality of life research

[18] Nilsen P (2015). Making sense of implementation theories, models and frameworks. Implement Sci.

[19] Varni JW, Seid M, Rode CA (1999). The PedsQL: measurement model for the pediatric quality of life inventory. Med Care.

[20] Qualitative Data Analysis Software | NVivo. Accessed November 15, 2021. https://www.qsrinternational.com/nvivo-qualitative-data-analysis-software/home

[21] Hsieh HF, Shannon SE (2005). Three approaches to qualitative content analysis. Qual Health Res.

[22] Black N (2013) Patient reported outcome measures could help transform healthcare. BMJ 346(7896). doi:10.1136/BMJ.F167 10.1136/bmj.f167 23358487

[23] Atkins L, Francis J, Islam R (2017). A guide to using the theoretical domains framework of behaviour change to investigate implementation problems. Implement Sci.

[24] Foster A, Croot L, Brazier J, Harris J, O’cathain A (2018) The facilitators and barriers to implementing patient reported outcome measures in organisations delivering health related services: a systematic review of reviews. J Patient Rep Outcomes. doi:10.1186/S41687-018-0072-3 10.1186/s41687-018-0072-3 PMC6170512 30363333

[25] Anthony SJ, Young K, Pol SJ, et al (2021) Patient-reported outcome measures in pediatric solid organ transplantation: exploring stakeholder perspectives on clinical implementation through qualitative description. Qual Life Res 30(5). doi:10.1007/S11136-020-02743-8 10.1007/s11136-020-02743-8 PMC8068689 33447959

[26] Philpot LM, Barnes SA, Brown RM (2018). Barriers and benefits to the use of patient-reported outcome measures in routine clinical care: a qualitative study. Am J Med Qual.

[27] Foster A, Croot L, Brazier J, Harris J, O’cathain A (2018) The facilitators and barriers to implementing patient reported outcome measures in organisations delivering health related services: a systematic review of reviews. J Patient-Rep Outcomes doi:10.1186/S41687-018-0072-3 10.1186/s41687-018-0072-3 PMC6170512 30363333

[28] Chawla N, Tom A, Sen MS, Sagar R (2021) Psychological impact of COVID-19 on children and adolescents: a systematic review. 43(4):294–299. doi:10.1177/02537176211021789 10.1177/02537176211021789 PMC8327877 34385721

