# Supplementary material for: Patient-reported outcome measures in pediatric asthma care: using theoretical domains framework to explore healthcare providers’ perceptions
Source: J Patient Rep Outcomes. 2022 Aug 19;6:88. doi: 10.1186/s41687-022-00494-3 (PMC9389517; doi:10.1186/s41687-022-00494-3)
Supplement: Supplementary file 1 — Additional file 1. Appendix 1: Interview Guide. [file 41687_2022_494_MOESM1_ESM.docx]

**Appendix 1: Interview guide for healthcare providers to identify barriers and enablers to PROM**

| **Theoretical Domain** | **Definition (Cane et al. 2012)** | **Sample Question** |
| --- | --- | --- |
| **Knowledge** | An awareness of the existence of something | 1. Have you ever heard about the use of patient reported outcome measures (PROMs) in routine clinical care?  2. Are you familiar with using any electronic tools to incorporate PROMs in clinical practice?  3. Are you familiar with the use of PROM? |
| **Skills** | An ability or proficiency acquired through practice | 1. In your opinion, what skills are needed to use PROM? (prompt –are there any other skills that you need?) |
| **Social/Professional Role & Identity** | A coherent set of behaviors and displayed personal qualities of an individual in a social or work setting | 1.What do you think is the purpose of using PROM?  2. Is providing incorporating PROMs in routine clinical care encouraged or discouraged by your professional association? |
| **Beliefs about Capabilities** | Acceptance of the truth, reality, or validity about an ability, talent or facility that a person can put to constructive use | 1. How easy or difficult would it be to incorporate PROMs in routine clinical care ? (prompt – what would make it easy or difficult for you?)  2. What challenges do you foresee encountering by incorporating PROMs in routine clinical care ?  3. How confident do you feel in your ability to use PROMs? |
| **Optimism** | The confidence that things will happen for the best or that desired goals will be attained | 1.How confident are you that patients will receive good care through the use of PROMs? |
| **Beliefs about Consequences** | Acceptance of the truth, reality or validity about outcomes of a behavior in a given situation | 1. What do you think are the consequences of providing care using PROMs? (negative)  2. What do you think are the benefits of incorporating PROMs in routine clinical care ?  3. Are there any harms in incorporating PROMs in routine clinical care?  4. What do you think is the balance of potential benefits and potential harms for incorporating PROMs in routine clinical? Does one outweigh the other? |
| **Reinforcement** | Increasing the probability of a response by arranging a dependent relationship, contingency, between the response and a given stimulus | 1. Are there any incentives for you to incorporate PROMs in routine clinical care? If yes, what are they?  2. What do you think will happen if PROMs are incorporate in your practice?  3. What do you think will happen if PROMs are NOT incorporated in your practice? |
| **Intentions** | A conscious decision to perform a behaviour or a resolve to act in a certain way | 1. On a scale of 1-10 and 10 being very important, how important do you think it is for you personally to incorporate PROMs in routine clinical care? |
| **Goals** | Mental representations of outcomes or end states that an individual wants to achieve | 1. Would the goal of incorporating PROMs in routine clinical care be compatible with your usual practice? (prompt – why?) |
| **Memory, Attention & Decision Processes** | The ability to retain information, focus selectively on aspects of the environment and choose between two or more alternatives | 1. Are there situations when you think it would be difficult to incorporate PROMs in routine clinical care? (prompt – can you tell me what is it about these situations that make it difficult)  2. What would help to overcome such situations? |
| **Environmental Context & Resources** | Any circumstance of a person’s situation or environment that discourages or encourages the development of skills and abilities, independence, social competence, and adaptive behavior | 1.What factors in your clinical environment would influence your decision to incorporate PROMs in routine clinical care? (prompt – to what extent do you feel these factors influence your ability)  2. What factors outside of your clinical environment would influence the use of PROMs in routine clinical care?  3. Are there competing tasks or time constraints that would influence your decision to incorporate PROMs in routine clinical care? |
| **Social Influences** | Those interpersonal processes that can cause individuals to change their thoughts, feelings, or behaviors | 1. Have you ever discussed the potential of incorporating PROMs in routine clinical care with other physicians or nurses in your care team? (Prompt- What was the summary of the discussion)  2. Would clinicians or any other member in your care team influence the use of PROMs? (prompt - How would they influence? To what extent?) |
| **Emotion** | A complex reaction pattern, involving experiential, behavioural, and physiological elements, by which the individual attempts to deal with a personally significant matter or event | 1. Does the idea of using PROMs evoke any emotional response in you? (prompt – would you feel worried or concerned about it?)  2. Would your patient’s emotions/preferences ever affect your decision of incorporating PROMs in routine clinical care? (prompt – what about patient’s families, would their emotions/preferences ever affect the decision of using PROM?) |
| **Behavioural Regulation** | Anything aimed at managing or changing objectively observed or measured actions | 1. What do you think is needed to ensure that we successfully implement PROMs in the asthma clinic ?  (prompt – things specific to you or your unit or your hospital) |
